# *Aedes aegypti* Aag-2 Cell Proteome Modulation in Response to Chikungunya Virus Infection

**DOI:** 10.3389/fcimb.2022.920425

**Published:** 2022-06-15

**Authors:** Anna Fernanda Vasconcellos, Reynaldo Magalhães Melo, Samuel Coelho Mandacaru, Lucas Silva de Oliveira, Athos Silva de Oliveira, Emily Caroline dos Santos Moraes, Monique Ramos de Oliveira Trugilho, Carlos André Ornelas Ricart, Sônia Nair Báo, Renato Oliveira Resende, Sébastien Charneau

**Affiliations:** ^1^ Laboratory of Biochemistry and Protein Chemistry, Department of Cell Biology, Institute of Biology, University of Brasilia, Brasilia, Brazil; ^2^ Laboratory of Virology, Department of Cell Biology, Institute of Biology, University of Brasilia, Brasilia, Brazil; ^3^ Laboratory of Toxinology and Center for Technological Development in Health, Oswaldo Cruz Foundation, Rio de Janeiro, Brazil; ^4^ Laboratory of Microscopy and Microanalysis, Department of Cell Biology, Institute of Biology, University of Brasilia, Brasilia, Brazil

**Keywords:** CHIKV, mosquito cell culture, label-free quantification, mass spectrometry, protein synthesis, apoptosis, RNA helicases, mitochondrion

## Abstract

Chikungunya virus (CHIKV) is a single-stranded positive RNA virus that belongs to the genus *Alphavirus* and is transmitted to humans by infected *Aedes aegypti* and *Aedes albopictus* bites. In humans, CHIKV usually causes painful symptoms during acute and chronic stages of infection. Conversely, virus–vector interaction does not disturb the mosquito’s fitness, allowing a persistent infection. Herein, we studied CHIKV infection of *Ae. aegypti* Aag-2 cells (multiplicity of infection (MOI) of 0.1) for 48 h through label-free quantitative proteomic analysis and transmission electron microscopy (TEM). TEM images showed a high load of intracellular viral cargo at 48 h postinfection (hpi), as well as an unusual elongated mitochondria morphology that might indicate a mitochondrial imbalance. Proteome analysis revealed 196 regulated protein groups upon infection, which are related to protein synthesis, energy metabolism, signaling pathways, and apoptosis. These Aag-2 proteins regulated during CHIKV infection might have roles in antiviral and/or proviral mechanisms and the balance between viral propagation and the survival of host cells, possibly leading to the persistent infection.

## Introduction

Chikungunya virus (CHIKV) is an Old World mosquito-borne virus that belongs to the genus *Alphavirus*, family *Togaviridae* (Cunha et al., 2020). This virus is transmitted to humans mainly by infected *Aedes aegypti* and *Aedes albopictus* blood meal bites, causing large epidemics worldwide (Lee et al., 2013). The word *chikungunya* means “the one that bends” in the Kimakonde African language (Azevedo et al., 2015) since CHIKV fever causes fatigue, rash, muscle pain, and severe polyarthralgia during the acute phase (World Health Organization, 2017). However, the infection can achieve a chronic stage, causing long-lasting painful and debilitating symptoms in the joints (Galán-Huerta et al., 2015). The lack of approved vaccines and specific antivirals against this arbovirus makes CHIKV fever an important public health issue (Cunha et al., 2020). Furthermore, environmental changes and the increasing number of world travelers in viremia also led to a significant expansion of CHIKV reaching area during the 2010s (Weaver, 2013). In Brazil, the first CHIKV autochthonous transmission occurred in 2014 in the Northern region, related to the Asian lineage. Soon after, the East–Central–South African (ECSA) lineage was detected in the Northeast, and it spread to all Brazilian regions in the following years, causing outbreaks in several states (Cunha et al., 2020).

CHIKV single-stranded positive RNA genome has approximately 11.8 kb, with polyA-tail and 5′ cap, and it exists as a single copy in each virion. It encodes two polyproteins that are cleaved in the course of infection by host and viral proteases (Strauss and Strauss, 1994). This generates four non-structural proteins 1–4 (nsP1–4) (Solignat et al., 2009) from the infectious genomic RNA, and five structural proteins (capsid, E3, E2, 6k, and E1) from a subgenomic RNA (Burt et al., 2017).

Virion entry in mammalian and Aedine cells can occur through clathrin-mediated endocytosis (Lee et al., 2013), and the surface protein receptor prohibitin has been associated with this process in human cells. Prohibitin has also been reported to facilitate *Dengue virus* 2 (DENV2) entrance in *Ae. aegypti* and *Ae. albopictus* cells (Kuadkitkan et al., 2010; Wintachai et al., 2012).

Both RNA replication and virion assembly take place in the cytoplasm of host cells (Strauss and Strauss, 1994). Non-structural proteins, such as RNA-dependent RNA polymerase (nsP4) and protease (nsP2), are translated before the structural ones (Kumar et al., 2021).

Structural proteins compose the ~65-nm-diameter, quasi-icosahedral, and enveloped CHIKV particle. E1/E2 glycoprotein dimers are arranged in 80 trimeric viral spikes on the mature virion membrane; while inside, the capsid is constituted by monomers of the capsid protein (CP) organized in a T = 4 geometry (Sharma et al., 2018). Spikes are essential structures for binding and fusion to the cell membrane. Since CHIKV can infect different types of cells, the use of multiple conserved receptors is required for recognizing and entering cells (Schnierle, 2019).

Historically, it has been extremely difficult to perform control of arbovirus vectors as a mitigation strategy (Achee et al., 2019). Thus, it is important to understand the biological mechanisms behind CHIKV persistent infection in *Aedes* spp. mosquitoes. Previous studies have addressed the proteomics of whole *Ae. aegypti* mosquitoes and their specific organs, such as midgut and salivary glands, using two-dimensional electrophoresis and mass spectrometry (MS) (Tchankouo-Nguetcheu et al., 2012; Shrinet et al., 2018; Cui et al., 2020; Chowdhury et al., 2021). The knowledge produced by proteomics may provide insights into molecular tools to diminish the vector competence for CHIKV. In this study, we aimed to perform a label-free quantitative proteomic analysis of CHIKV-infected *Ae. aegypti* Aag-2 cells to give an overview of the protein abundance profile changes in time points 0, 12, and 48 h postinfection (hpi). Proteomics and microscopy data revealed several regulated proteins that are possibly involved in host cell metabolic shifts that could assist viral replication.

## Material and Methods

### Cells and Virus

*Ae. aegypti* Aag-2 cells (RRID : CVCL_Z617) were kindly provided by Gorben Pijlman, PhD (Wageningen University & Research, Netherlands) and cultured in Schneider’s medium (Sigma-Aldrich, St. Louis, MO, USA) supplemented with 10% fetal bovine serum (FBS) and 100 U/ml of penicillin/streptomycin at 28°C. *Ae. albopictus* C6/36 cells were maintained in TC-100 medium (Vitrocell Embriolife, Campinas, Brazil) supplemented with 10% FBS at 28°C. African green monkey kidney-derived Vero cells were grown in high-glucose Dulbecco’s modified Eagle‘s medium (DMEM; Sigma-Aldrich) supplemented with 10% FBS and 100 U/ml of penicillin/streptomycin in an incubator at 37°C under 5% CO_2_. Cell passages were performed once a week. The CHIKV isolate (strain Cuiabá-MT, Brazil, National Center for Biotechnology Information (NCBI) accession number MH823667) was supplied by the Central Public Health Laboratory of the Federal District (LACEN-DF).

### Virus Recovery and Propagation

The CHIKV isolate was recovered from an infected patient’s blood sample and inoculated in Vero cells, which present a clear cytopathic effect (CPE) during CHIKV infection. Three days postinfection (dpi), the onset of CPE was noted, and the supernatant was collected. Viral RNA was extracted from Vero cells using TRIzol (Thermo Fisher Scientific, Waltham, MA, USA), and replication was confirmed by RT-PCR. SuperScript IV reverse transcriptase (Thermo Fisher Scientific) was used for cDNA synthesis, while PCR was performed with Platinum Taq DNA polymerase (Thermo Fisher Scientific). The primers forward 5′-CGAAGAGTGGAGTCTKGCATYCCAG-3′ and reverse 5′-GCCTCYTGGTATGTGGCCGCTTTAGC-3′ amplified the set of E3–E2 genes, generating an amplicon about 1.5 kb. Then, *Ae. albopictus* C6/36 cells were infected with virus-containing supernatant from Vero culture to increase the virus titer, since these mosquito cells have a defective antiviral RNAi machinery, being advantageous for arbovirus propagation (Brackney et al., 2010). C6/36 cell culture supernatant was collected for the following steps.

### Chikungunya Virus Titration

Viral titer was obtained by End-Point Dilution Assay in Terasaki plates. Confluent Vero cells grown in a T25 flask were detached with 1 ml of trypsin-EDTA and diluted in 4 ml of DMEM supplemented with 10% FBS and 100 U/ml of penicillin/streptomycin. The cells in suspension were incubated with serial dilutions (from 1 × 10^−1^ to 1 × 10^−9^) of virus stocks in a 1:1 volume ratio (90 µl cell suspension:90 µl virus dilution). In 60-well Terasaki plates (Greiner Bio-One, Kremsmünster, Austria), 10 µl per well was added in 6 replicates per dilution. The CHIKV titration was achieved based on CPE visualized by light microscopy at 3 dpi and TCID50 calculation. Each condition was titrated twice, and the mean number of viral particles was considered.

### Growth Kinetics in Aag-2 Cells

To explore changes in CHIKV-infected Aag-2 cells at the protein level, the culture harvesting times were determined according to the highest virus titer. Multiplicities of infection (MOIs) of 0.1 and 1 were used to infect Aag-2 cells in 6-well plates in biological triplicates. After 90 min, the supernatant was replaced with fresh Schneider’s medium supplemented with FBS and 100 U/ml of penicillin/streptomycin. The CHIKV-infected supernatants were titrated at 24, 48, and 72 hpi by End-Point Dilution Assay. The virus growth curve was set, and the harvesting time points 0, 12, and 48 hpi were determined for the following experiments, with a chosen MOI of 0.1.

### Transmission Electron Microscopy

Aag-2 cells were grown in three independent T25 flasks and cultured in Schneider’s medium (Sigma-Aldrich) supplemented with 10% FBS and 100 U/ml of penicillin/streptomycin at 28°C. Each flask was harvested at a different time point: 0 (uninfected control), 12, and 48 hpi. The samples were transferred to 15-ml tubes and centrifuged at 1,200 ×*g* for 3 min. After two rounds of phosphate-buffered saline (PBS) washing, pellets were transferred to microtubes, and fixation occurred overnight at 4°C in a solution containing 2% (v/v) paraformaldehyde, 2% (v/v) glutaraldehyde, and 0.1 M of sodium cacodylate buffer, pH 7.2. Next, whole preparations were post-fixed, for 30 min in 2% (w/v) osmium tetroxide, 1.6% potassium ferricyanide, and 10 mM of CaCl_2_ in 0.2 M of sodium cacodylate buffer, pH 7.2. Samples were washed in distilled water and stained in a block with 0.5% (w/v) uranyl acetate for 24 h at 4°C. The material went through dehydration in a graded acetone series (50%–100%) for 10 min each, and it was embedded in Spurr resin. Ultrathin sections were obtained using an ultramicrotome UC6 (Leica, Wetzlar, Germany) that were contrasted with uranyl acetate and lead citrate. The final step was to examine and photograph the sections under a Jeol 1011 transmission electron microscope (TEM; Jeol 1011, Tokyo, Japan) at 80 kV.

### Sample Preparation for Mass Spectrometry

In T25 flasks, 2 × 10^6^ Aag-2 cells were seeded. After 16 h, cells were infected with CHIKV stock at MOI of 0.1 in biological triplicates. Mock cells (with Schneider’s medium only) were harvested at t = 0 h (uninfected), and CHIKV-infected cells were harvested at 12 and 48 hpi. Aag-2 pellets were lysed with 8 M of urea in 20 mM of ammonium bicarbonate, pH 7.9, containing a complete mixture of protease and phosphatase inhibitors (Roche, Basel, Switzerland). Then, 100 μg of protein was reduced with 5 mM of dithiothreitol (DTT) for 1 h at 32°C and alkylated with 14 mM of iodoacetamide for 40 min at room temperature in the dark. Samples were then applied to a filter (Centrifugal Filters Ultracel, 3 kDa) and centrifuged for 10 min at 14,000 ×*g*. Next, a washing step was performed with 8 M of urea followed by centrifugation for 10 min at 14,000 ×*g*. A total of 2 μg of modified trypsin (Promega, Madison, WI, USA) was used for tryptic digestion following the proportion ratio of 1:50 (enzyme:substrate) for 18 h at 37°C under 600-rpm agitation. Tryptic peptides were acidified by adding trifluoroacetic acid (TFA) to a final concentration of 0.1% (v/v), desalted with POROS R2 resin (Applied Biosystems, Foster City, CA, USA), and packaged in micropipette tips. Desalted peptides were vacuum dried and suspended in 0.1% (v/v) formic acid (FA), and aliquots corresponding to 0.5 µg/µl were used for MS analysis.

### Mass Spectrometry

Tryptic digests were analyzed by reversed-phase nanoscale liquid chromatography coupled to high-resolution nanoelectrospray ionization MS. Chromatography was performed using a Dionex Ultimate 3000 RSLCnano system coupled to the HF-X Orbitrap MS (Thermo Fisher Scientific). All samples (1 μg per run) were initially applied to a 2-cm guard column, followed by fractionation on a 25.5-cm PicoFrit™ Self-Pack column (New Objective) packed with 1.9-μm silica, ReproSil-684 Pur 120 Å C18-AQ (Dr. Maisch, Ammerbuch, Germany). Each sample was loaded in 0.1% (v/v) FA and 2% (v/v) acetonitrile (ACN) onto the trap column at 2 μl/min, while chromatographic separation occurred at 200 nl/min. Mobile phase A consisted of 0.1% (v/v) FA in water, while mobile phase B consisted of 0.1% (v/v) FA in ACN. Peptides were eluted with a linear gradient from 2% to 40% eluent B over 32 min, followed by up to 80% B in 4 min. Lens voltage was set to 60 V. Full-scan MS mode was acquired with a resolution of 60,000 (FWHM at *m*/*z* 200 and automatic gain control (AGC) set to 3 × 10^6^). The 20 most abundant precursor ions from each scan (*m*/*z* 350–1,400) were sequentially subjected to fragmentation by higher-energy collisional dissociation (HCD). Fragment ions were analyzed at a resolution of 15,000 using an AGC set to 1 × 10^5^. Data were acquired using Xcalibur software (version 4.2.47).

### Label-Free Protein Quantification and Identification

Protein quantification and identification were performed by using MetaMorpheus (Solntsev et al., 2018), which takes advantage of a modified version of Morpheus (Wenger and Coon, 2013) for identification and FlashLFQ (Millikin et al., 2017) for quantification. Briefly,.raw files and a FASTA file containing the UniProt reference proteomes of CHIKV (UP000000569) and *Ae. aegypti* (UP000008820), with one protein sequence per gene, were loaded into MetaMorpheus. Both FASTA files were downloaded in May 2021. Then, calibration was performed with default values, with file-specific tolerances set to each MS run. Next, global post-translational modification discovery (G-PTM-D) was used to search for common biological, common artifacts, and metal modifications in the files. Identification and quantification were done allowing a maximum of two missed cleavages, two modifications per peptide, and a peptide minimum length of 7. Carbamidomethylation of cysteine and selenocysteine was set as fixed modifications. Oxidation of methionine was set as a variable modification. Protein parsimony was applied, two peptides were required to identify a protein group, and modified peptides were treated as different peptides to remove ambiguities in the quantification of proteins. Quantification was performed without match between runs (MBR), and results were normalized by FlashLFQ. Peptide-spectrum matches (PSMs) were considered valid if the q-value <0.01 and the MetaMorpheus minimum score was set to 5.

### Quality Control, Statistical Analysis, and Functional Annotation

The resulting protein groups were imported into the R environment (R Core Team, 2019), within RStudio (RStudio Team, 2021). The quality control and statistical analysis were performed using pmartR (Stratton et al., 2019) with the assistance of the following packages: heatmaply (Galili et al., 2017), dendextend (Galili, 2015), ggplot2 (Wickham, 2016), and GAGE (Luo et al., 2009). First, proteins with less than two unique peptides were filtered out, and protein abundance was log2 transformed. Subsequently, filters were applied according to the number of missing values. Proteins were required to have at least three quantified values in the total replicates and two quantified values per group. The resulting protein groups were analyzed by robust Mahalanobis distance using correlations, median absolute deviations, skewness, and proportion of missing values to verify the presence of extreme outliers. ANOVA and *post-hoc* Bonferroni multiple test correction with a p-value <0.05 cutoff were used to identify proteins and peptides regulated between groups. CHIKV peptides were related to the mature proteins through a comparison of their position in the CHIKV protein sequence and annotation of the structural polyprotein (Q8JUX5) of the CHIKV strain S27-African prototype. Variance-sensitive clustering through VSClust (Schwämmle and Jensen, 2018) and heatmap clustering were done by using proteins without missing values. Furthermore, the optimal number of clusters was defined by minimum centroid distance. To perform enrichment analysis, UniProt protein AC was converted to Entrez GeneID using UniProtKB (The UniProt Consortium, 2021) and db2db (Mudunuri et al., 2009). The resulting 984 Entrez GeneID with their corresponding abundances were analyzed by Generally Applicable Gene-set Enrichment (GAGE) against the Kyoto Encyclopedia of Genes and Genomes (KEGG) gene set of *Ae. aegypti*. Pathways were considered enriched if q-value <0.1. Protein-protein interaction (PPI) network was constructed by using STRING (Szklarczyk et al., 2019) without text mining, and Cytoscape StringApp (Doncheva et al., 2019) with the help of yfiles layout algorithms app.

## Results

**Figure 1:**
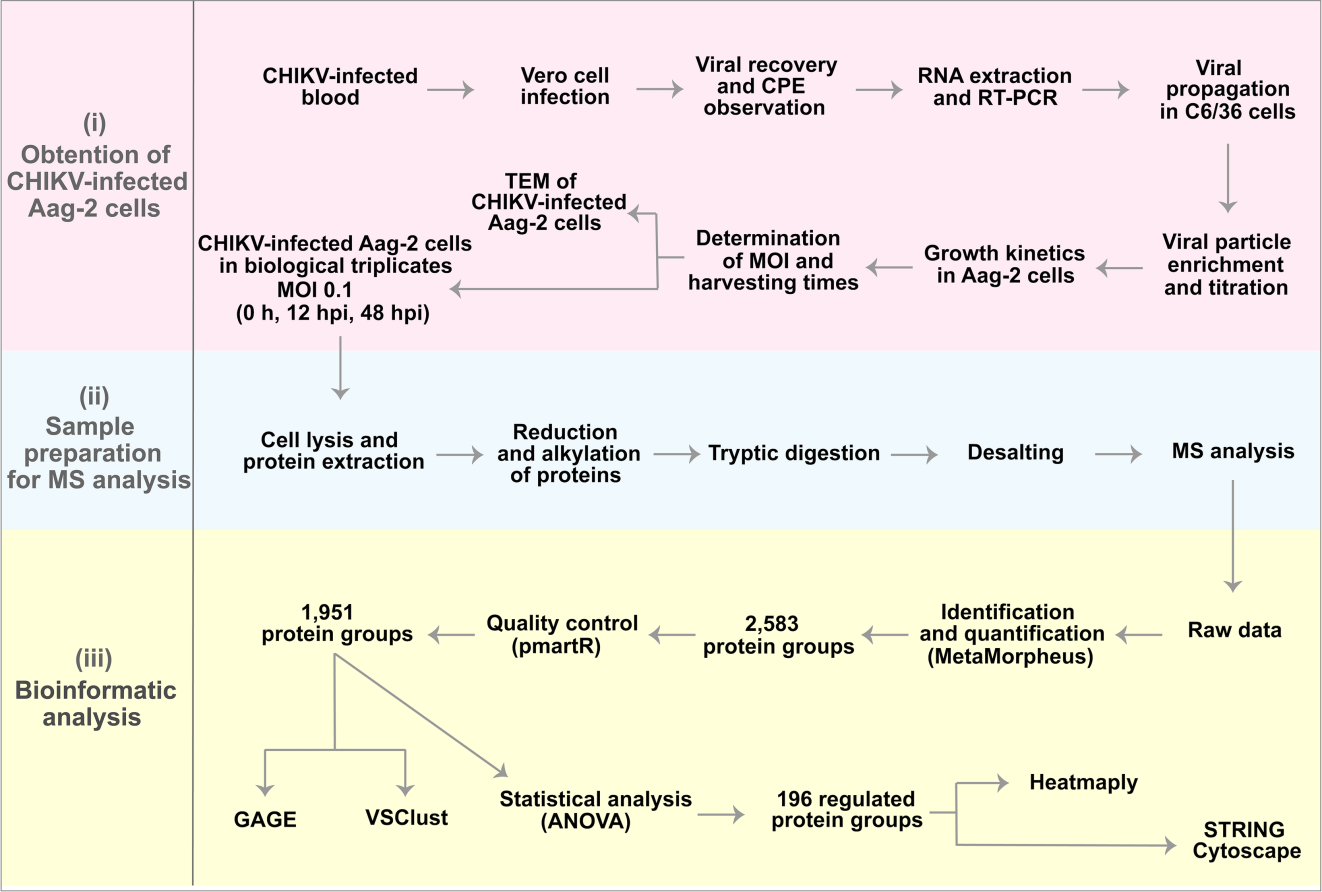
Experimental design and preliminary results. (i) Obtention of chikungunya virus (CHIKV)-infected Aag-2 cells, which consisted of the determination of multiplicity of infection (MOI) (1), harvesting times (0, 12, and 48 hpi), and microscopy analysis. (ii) Sample preparation for mass spectrometry (MS) analysis. (iii) Bioinformatics analysis that resulted in 196 proteins statistically significant as upregulated or downregulated.

To study the *Ae. aegypti* Aag-2 cell response to CHIKV infection, we designed an experimental label-free quantitative proteomic procedure supported by microscopy analysis (**Figure 1**).

### Determination of Multiplicity of Infection and Harvesting Time Points for Chikungunya Virus-Infected Aag-2 Cells

A growth kinetics curve in infected Aag-2 cells was performed to identify the time point with the highest production of viral particles and the most suitable CHIKV MOI for *Ae. aegypti* cells. Initially, we performed a CHIKV infection using MOI of 1 in Vero cells, since those cells allow a distinguishable CPE. As infection control, we used isolates of DENV2 and *Mayaro virus* (MAYV), also using MOI of 1. As a negative control, we used mock-infected Vero cells. From these assays, we could notice that our CHIKV isolate was more virulent than the other arboviruses, producing higher levels of CPE (syncytia formation) and cell death (**Supplementary Figure 1**). The CHIKV isolate (NCBI accession number MH823667) used in this study was sequenced and phylogenetically clustered with isolates of the ECSA (Vasconcellos et al., 2019). This lineage is known to be more virulent than other CHIKV lineages since ECSA isolates can interfere with the innate immune system by impairing interferon activation (Figueiredo and Figueiredo, 2014; Langsjoen et al., 2018). For this reason, CHIKV MOIs of 0.1 and 1 were tested to infect Aag-2 cells to establish growth kinetics.

**Figure 2:**
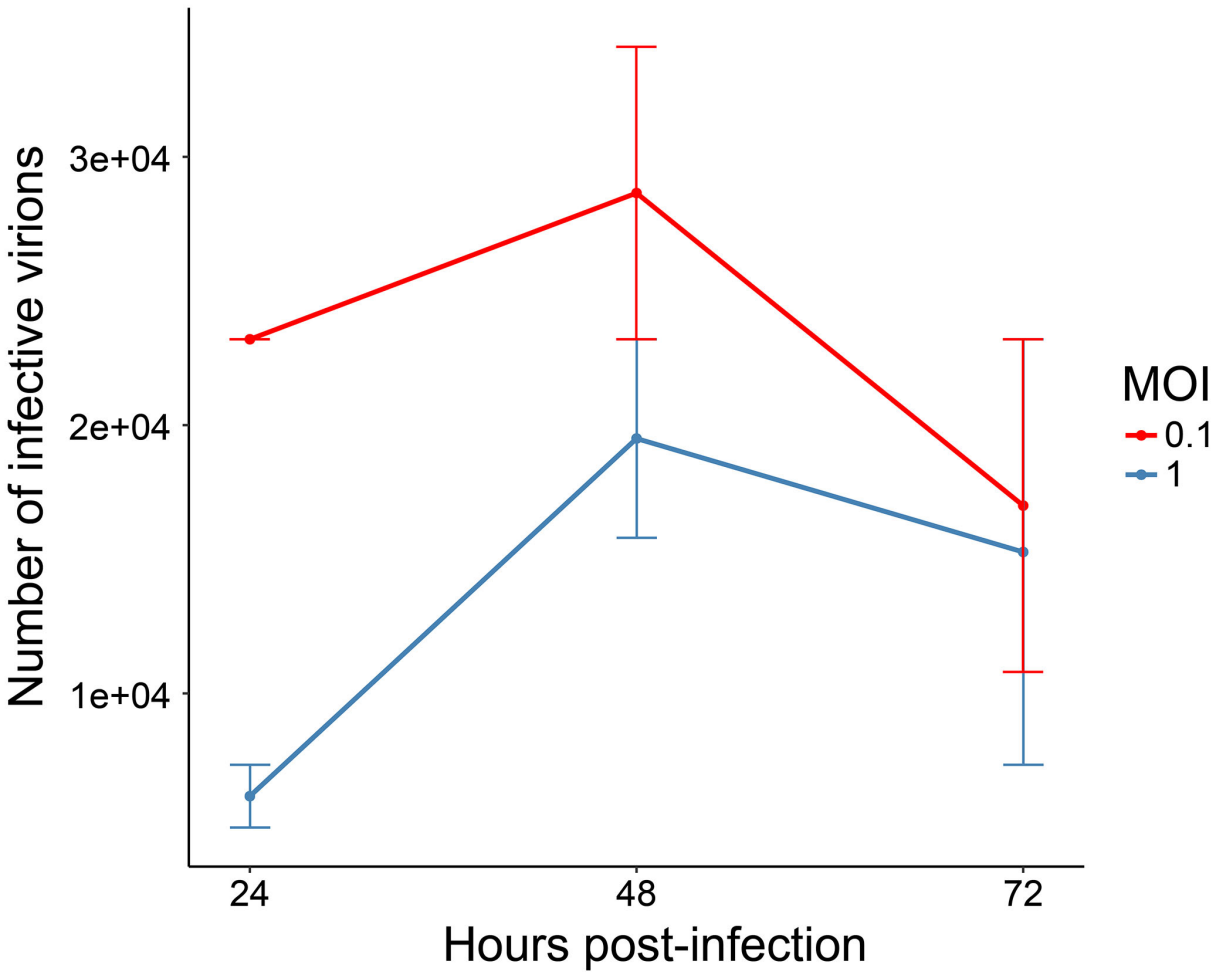
Growth kinetics of chikungunya virus (CHIKV)-infected Aag-2 cells. The multiplicities of infection (MOIs) of 0.1 and 1 were used to infect mosquito cells, and the supernatant was collected at three different time points (24, 48, and 72 hpi). Titration of infective CHIKV particles was performed with EndPoint Dilution Assay. The chosen MOI was 0.1, and the harvesting time points 12 and 48 hpi were selected.

We determined the times 24, 48, and 72 hpi to harvest supernatant and perform viral titration. As expected, the growth kinetics showed a higher CHIKV particle production at all harvesting times using MOI of 0.1, which was chosen for the next steps. Supernatant titration showed a slightly higher viral particle production at 48 hpi (2.86 × 10^4^/ml) rather than at 24 hpi (2.32 × 10^4^/ml) and at 72 hpi (1.7 × 10^4^/ml) (**Figure 2**). Very similar results were obtained in a previous work, when CHIKV-infected Aag-2 cells achieved higher titrations at 48 hpi, using both MOIs of 0.1 and 1 (Kumar et al., 2021). Accordingly, 48 hpi was established as the final harvesting time point. In addition, considering the enhanced virulence aspect of our CHIKV isolate, we also decided to choose an intermediate harvesting time point of 12 hpi. An earlier harvesting time point could shed light on interesting biological aspects in the protein abundance of CHIKV-infected Aag2 cells, presented in our proteomic analysis (see below). Therefore, we established the CHIKV MOI of 0.1 to infect *Ae. aegypti* cells, for further analysis at 0 (uninfected), 12, and 48 hpi.

### Transmission Electron Microscopy of Chikungunya Virus-Infected Aag-2 Cells

**Figure 3:**
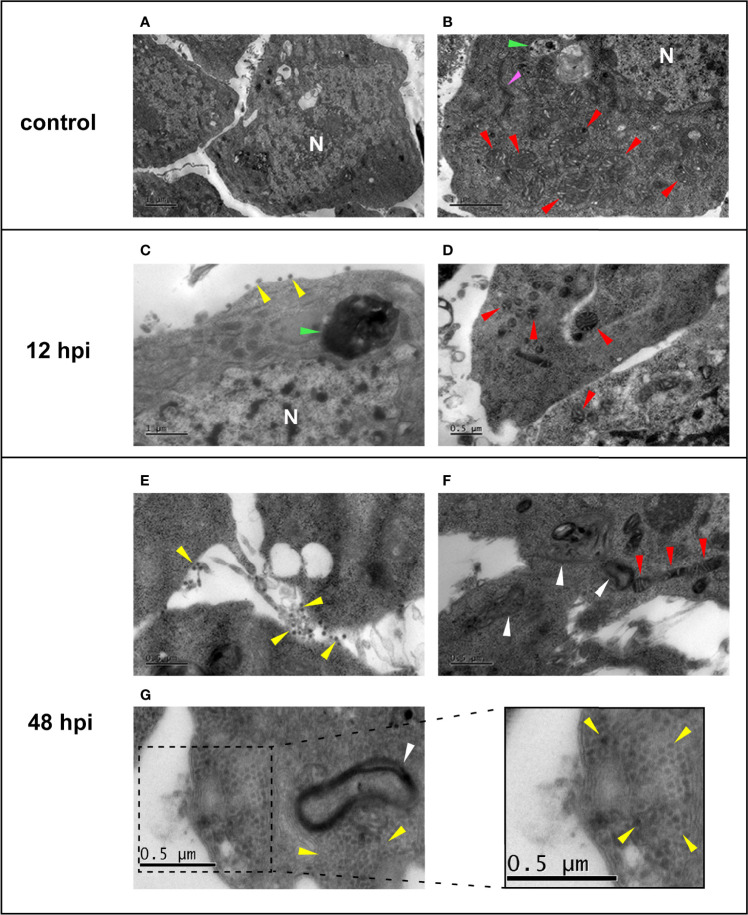
Electron micrographs of uninfected **(A, B)** and chikungunya virus (CHIKV)-infected **(C–G)** Aag-2 cells. **(A, B)** Uninfected cells showing the mitochondria (red arrows), autophagic vesicle (green arrow), a possible Golgi complex (pink arrow), and the nucleus (N). **(C, D)** Aag-2 cells infected with CHIKV using multiplicities of infection (MOIs) of 0.1 at 12 hpi. **(C)** Possible released viral particles can be seen in the extracellular space (yellow arrows), and the proposed autophagic vesicle is darker (green arrow). Mitochondria (red arrows) and the nucleus (N) membranes are apparently still preserved. **(E–G)** Aag-2 cells infected with CHIKV at 48 hpi using MOI of 0.1 showing possible virions (yellow arrows) found in both extracellular **(E)** and intracellular spaces **(G)**, inset, while mitochondria present a suggestive abnormal elongated morphology (red arrows) **(F)**, and membrane residues are observed (white arrows), probably associated with cytopathic effect (CPE) **(F, G)**. Bars represent 1 μm in **(A–C)** and 0.5 μm in all other images.

TEM analysis was performed using the established MOI of 0.1 and the harvesting time points 0, 12, and 48 hpi, to visually support the biological effects of CHIKV infection. Uninfected Aag-2 cells (Control) presented a well-defined morphology, with apparently intact mitochondria, nucleus, autophagosome vesicle, and a structure that appears to be a Golgi complex (**Figures 3A, B**). At 12 hpi, a small number of viral particles in the extracellular space were observed, although the integrity of the plasmatic membrane and organelles, such as the nucleus and mitochondria, seem to remain preserved (**Figures 3C, D**). Conversely, an apparent CPE is observed in Aag-2 cells at 48 hpi. Degraded fragments of membranes can be seen, possibly from cytoplasmic membranes and membranous organelles (**Figures 3E, F**). Moreover, mitochondria present a suggestive abnormal elongated morphology at 48 hpi (**Figure 3F**). Considering our TEM images, viral particles can be found in both intracellular and extracellular environments. However, a surprisingly elevated number of virions in the intracellular space of the mosquito cell at 48 hpi are observed in **Figure 3G**. According to our observations, despite that viral production had already been verified at 12 hpi, a significant cell integrity loss and virion production could be only noticed at 48 hpi, in agreement with the growth kinetics (**Figure 2**). This pattern is also observed for other arboviruses, such as MAYV (Vasconcellos et al., 2020).

### Proteomic Analysis

A total of 2,583 protein groups with at least two unique peptides were identified (**Supplementary Table 1**). The efficiency of the normalization of protein group abundances was assessed by boxplot and histogram, exhibiting a bell-shaped distribution (**Supplementary Figures 2A, B**). We also applied filters to ensure the use of more confident protein groups in quantifications. First, protein groups with more than three missing values in the total replicates were removed, resulting in 2,296 protein groups (**Supplementary Figure 2C**). Then, to perform ANOVA, protein groups with less than two quantified values per condition were removed, resulting in 1,951 protein groups (**Supplementary Figure 2D**). These protein groups were submitted to robust Mahalanobis distance analysis to evaluate the presence of extreme outliers, and none could be observed (**Supplementary Figure 2E**). Moreover, we performed a probabilistic principal component analysis (PPCA) to demonstrate the grouping of the replicates among conditions (**Supplementary Figure 2F**).

**Figure 4:**
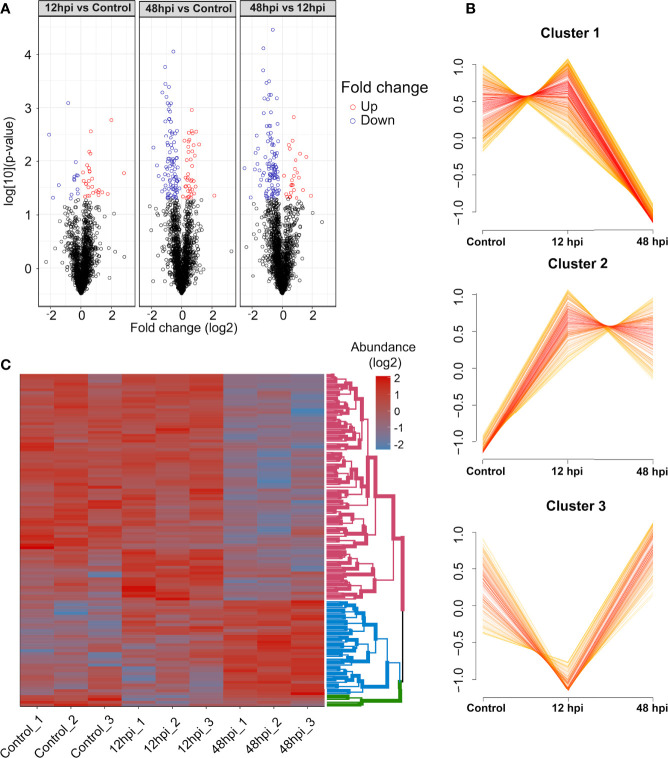
Quantitative overview of abundance variation of protein groups during chikungunya virus (CHIKV) infection of *Aedes aegypti* Aag-2 cells. **(A)** Volcano plot highlighting regulated protein groups analyzed by one-way ANOVA with *post-hoc* Bonferroni multiple test correction (p-value < 0.05) from 1,951 protein groups analyzed. **(B)** Clusters of abundance profiles of 1,129 protein groups were obtained in VSClust. **(C)** Heatmap of 116 protein groups without missing values clustered according to their abundance patterns.

Next, the 1,951 protein groups were tested by one-way ANOVA, which resulted in 196 protein groups with a difference in the abundance quantification between the conditions analyzed (**Supplementary Table 2**). By comparing the abundance fold-change (FC) of these protein groups observed between 12 hpi vs. Control, 48 hpi vs. Control, and 48 vs. 12 hpi, it is possible to notice that the 12 hpi vs. Control comparison presents a fewer number of differentially abundant protein groups (**Figure 4A**). Conversely, the other two comparisons, 48 hpi vs. Control and 48 vs. 12 hpi, presented a greater number of regulated protein groups, comprising 130 and 128, respectively. Moreover, a variance-sensitive clustering (VSClust) analysis was performed, resulting in three clusters of *Ae. aegypti* protein groups. This analysis clustered 738 protein groups, comprising 365, 215, and 158 protein groups, in clusters 1, 2, and 3, respectively (**Supplementary Table 3**). Protein abundance in cluster 1 decreased along with the 48 hpi compared to Control (**Figure 4B**). Conversely, in cluster 2, the abundance during the course of infection (12 and 48 hpi) was greater than in Control. Cluster 3 showed a lower abundance at 12 hpi and an abundance recovery at 48 hpi.

From those 196 regulated protein groups, tested by one-way ANOVA followed by Bonferroni multiple test correction, 116 did not present any missing values. These protein groups were submitted to clustering in a heatmap analysis (**Figure 4C**). Consistently with VSclust analysis (**Figure 4B**), two consistent patterns of protein modulation in the CHIKV-infected Aag-2 cells are exhibited, especially considering clusters 1 (pink nodes) and 2 (blue nodes): i) predominantly decreased abundance the last time postinfection (48 hpi) compared to Control and 12 hpi and ii) increased abundance in the last time postinfection (48 hpi) compared to Control and 12 hpi. In contrast, cluster 3 presented a different abundance pattern. Protein abundance abruptly decreased at 12 hpi compared to the control and drastically increased from 12 to 48 hpi. Despite 158 protein groups in cluster 3, only four protein groups were present in the heatmap analysis (**Figures 4B, C**).

### Abundance of Chikungunya Virus Peptides Throughout Infection

**Figure 5:**
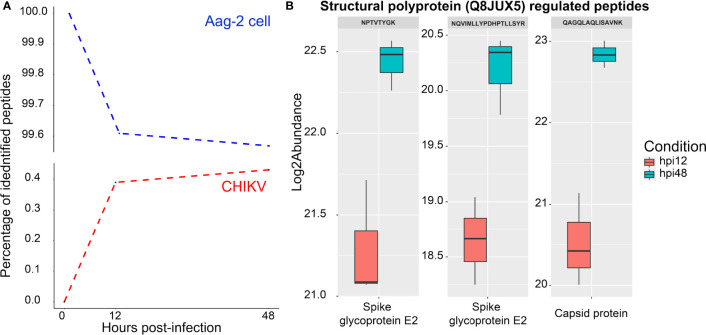
Analysis of chikungunya virus (CHIKV) and *Aedes aegypti* peptides during the infection. **(A)** Percentage of *Aedes Aegypti* (blue dotted line) and CHIKV (red dotted line) identified peptides over the time points (0, 12, and 48 hpi). **(B)** Regulated CHIKV peptides between 12 and 48 hpi, which correspond to the E2 spike glycoprotein and the capsid protein. Those proteins are proteolytically cleaved from the structural polyprotein (Q8JUX5).

Since growth kinetics and TEM showed an increase of viral particles until 48 h (**Figure 2**), the percentage of identified CHIKV peptides was expected to increase during the course of infection, as observed in **Figure 5**, reaching only about 0.4% of all identified peptides at 48 hpi. It is noteworthy to mention that an MOI of 0.1 was used to infect Aag-2 cells. A previous study developed by our group used MOI of 1 to infect mosquito cells with MAYV, and a higher percentage of viral peptides was observed (Vasconcellos et al., 2020).

Two CHIKV proteins were identified at 12 and 48 hpi, referring to P1234 (Q8JUX6) and structural (Q8JUX5) polyproteins. None of them were found to be regulated. Both polyproteins are proteolytically cleaved along the viral infectious cycle into non-structural (nsP1–4) and structural proteins (CP, E3, E2, 6k, and E1) (Solignat et al., 2009). Regarding CHIKV peptides, three of them were differentially abundant (**Figure 5B**), all part of the structural polyprotein (Q8JUX5). Two peptides (NPTVTYGK and NQVIMLLYPDHPTLLSYR) were identified in the region of the spike glycoprotein E2 and one (QAGQLAQLISAVNK) as part of the CP (**Figure 5B**).

### Bioinformatics

**Figure 6:**
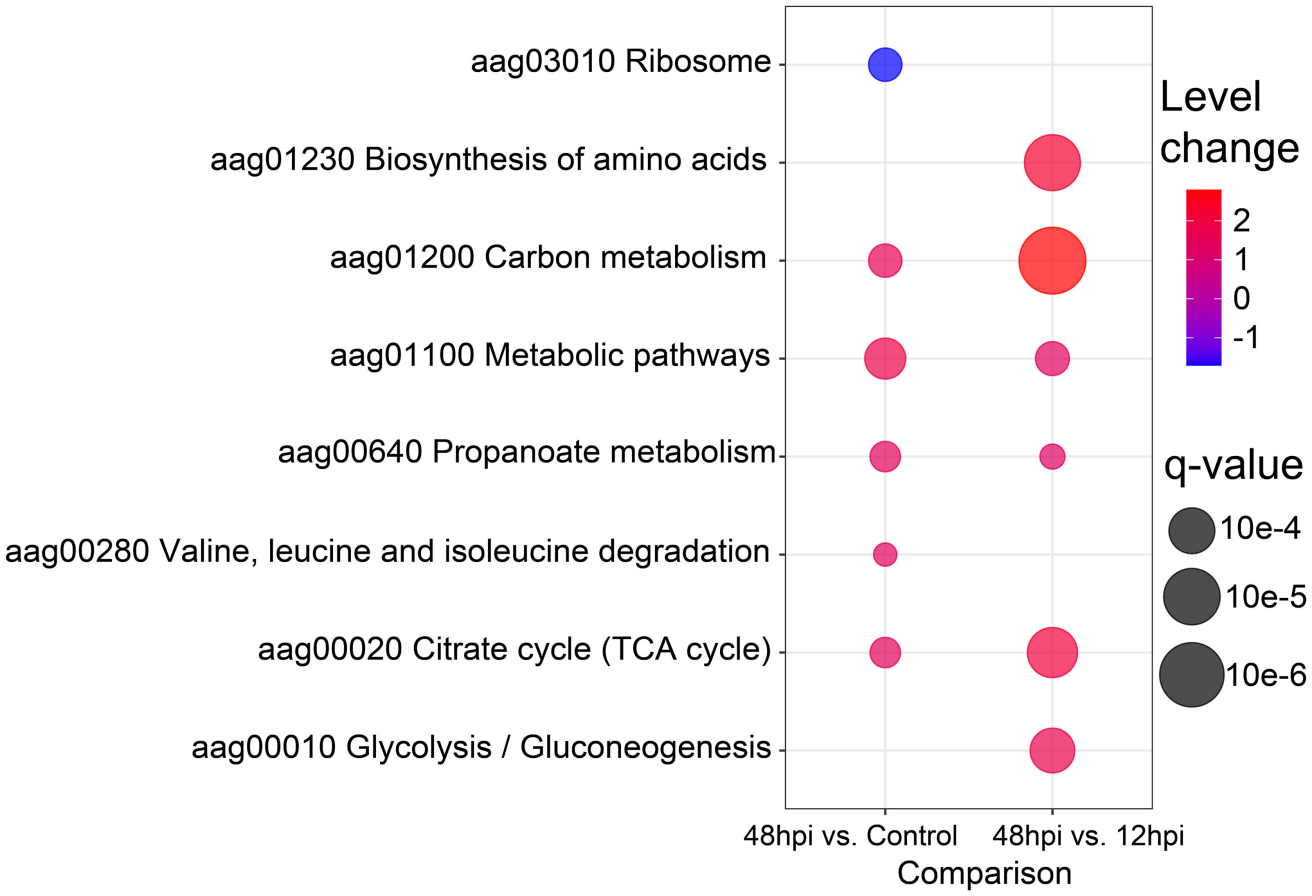
Bubble plot of enriched Kyoto Encyclopedia of Genes and Genomes (KEGG) pathways. The eight enriched KEGG pathways by Generally Applicable Gene-set Enrichment (GAGE) analysis (q-value < 0.1) are shown in a bubble plot according to their q-values and level changes, where level changes represent the magnitude of KEGG pathway variation, and q-value the significance for each gene-set enrichment.

Volcano plot (**Figure 4A**) and PPCA (**Supplementary Figure 2F**) showed a more similar abundance pattern of protein groups between 12 hpi and the uninfected condition, rather than 48 hpi. In agreement, no enriched pathway was found between the 12 hpi vs. Control by GAGE enrichment analysis (data not shown), while the time point comparisons encompassing 48 hpi displayed a difference in the protein abundance related to several pathways. Eight KEGG sets were enriched: ribosome (aag03010), biosynthesis of amino acids (aag01230), carbon metabolism (aag01200), metabolic pathways (aag01100), propionate metabolism (aag00640), valine, leucine and isoleucine degradation (aag00280), citrate cycle (tricarboxylic acid (TCA) cycle) (aag00020), and glycolysis/gluconeogenesis (aag00010) (**Figure 6**). Interestingly, the ribosome (aag03010) set was the only gene set to present a negative level change, suggesting the presence of proteins more abundant in the Control compared to 48 hpi. In contrast, carbon metabolism was enriched in both comparisons, with a great positive level change in 48- compared to 12-hpi comparison. Moreover, other pathways presented a positive level change in both comparisons, and some of these sets could be related to energy metabolic processes, such as the citrate cycle (TCA cycle) (aag00020), glycolysis/gluconeogenesis (aag00010), and metabolic pathways (aag01100) (**Figure 6**). These observations suggest an enhanced carbon metabolism and a decreased abundance in proteins related to ribosomes at 48 hpi.

**Figure 7:**
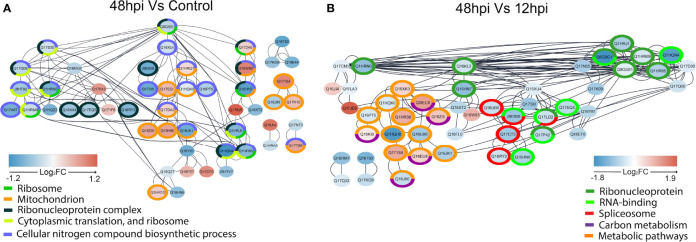
STRING network interaction analysis of regulated proteins in 48 hpi vs. Control **(A)** and 48 vs. 12 hpi **(B)**. Interactions of regulated proteins analyzed by one-way ANOVA with *post-hoc* Bonferroni multiple test correction (p-value < 0.05) were assessed by STRING without text mining and imported to the Cytoscape, where overrepresentation analysis of biological terms was performed. The overrepresented terms are indicated by different colors in the legends, and the proteins that belong to these terms are circled in the respective colors. Log_2_FC are represented by filled colors (red for upregulated and blue for downregulated), and smaller node distances correspond to higher protein–protein interaction scores.

Regulated proteins at 48 hpi compared to Control (**Figure 7A**) and at 48 compared to 12 hpi (**Figure 7B**) were submitted to STRING analysis to predict the PPI network to highlight regulated biological processes. The main biological terms encountered in the PPI clusters were related to carbon metabolic processes and protein synthesis as observed by GAGE analysis (**Figure 6**). Overall, in the two pairwise comparisons, PPI clusters with Gene Ontology (GO) terms related to energetic metabolism presented predominantly positive FC in the PPI network at 48 hpi. Conversely, PPI clusters with terms related to protein synthesis were mostly represented by proteins with lower FC in the PPI network at 48 hpi.

## Discussion

### *De Novo* Synthesis of the Virus Over 48 h

Higher demand for structural proteins for *de novo* synthesis of virions could explain the progressive detection of viral structural components over 48 h (**Figures 3**, **5**). In an alphaviral particle, 240 monomers of CP protein are structured together to form the capsid, in which about 80 trimeric E1/E2 proteins called spikes are inserted (Brown et al., 2018). Therefore, 240 CP and approximately 240 E2 proteins (80 ×3) are required to assemble a single CHIKV particle. E2 belongs to the immunoglobulin superfamily, and its cytosolic domain interacts with CP; thus, E2-CP interaction is needed for viral particle assembly and budding (Cunha et al., 2020). CP itself is responsible for the packaging of viral genomic RNA. Together, they form the nucleocapsid (Brown et al., 2018).

### Protein Synthesis Inhibition of Chikungunya Virus-Infected Aag-2 Cells at 48 hpi

Since viruses are intracellular parasites that carry only their genomes and specific proteins; they depend on recruiting several host proteins to replicate, such as ribosomal proteins (RPs) (Petit and Shah, 2019). RPs may cause different effects in a virus life cycle: enhancing viral proliferation, translating viral proteins, or even inhibiting viral infection. Usually, after viral infection, host mRNA translation is suppressed (Li, 2019). In this study, we could identify several proteins related to protein syntheses, such as proteins of Spliceosome, Ribonucleoprotein complex, Cytoplasmic translation, and Ribosome. These proteins were predominantly downregulated over 48 h in all analyses performed. Specifically, in VSclust analysis, cluster 1 proteins were overrepresented with GOs related to protein synthesis (**Supplementary Figure 3**), while differentially abundant proteins related to protein metabolism are present in the PPI networks (**Figure 7** **),** and the enrichment analysis of the KEGG pathway ribosome gene set is enriched with negative level change at 48 hpi (**Figure 6**). Previous reports revealed a widespread translational shutoff of human fibroblasts in the late stages of CHIKV infection (White et al., 2011). Despite the translational shutoff, the translation of CPs remained active (White et al., 2011). Moreover, Cui et al. (2020) observed that CHIKV-infected *Ae. aegypti* mosquitoes had protein synthesis perturbed/inhibited from 24 to 96 hpi. Other viruses, such as poxvirus, influenza viruses, and hantaviruses, are also known to inhibit host protein synthesis. In these cases, inhibition is performed by affecting the cap of host mRNA and its interactions with eukaryotic initiation factor 4 (eIF4). On the other hand, measles virus, rabies virus, and foot-and-mouth disease virus target eukaryotic initiation factor 3 (eIF3), also resulting in host protein translation inhibition (Walsh and Mohr, 2011). In the present study, we detected three downregulated subunits of the eIF3 at 48 hpi when compared to 12 hpi and/or Control: eIF3 subunit C (Q17Q06, FC 48 hpi vs. Control −0.43, Cluster 1), eIF3 subunit M (Q17D30, FC 48 hpi vs. Control −0.28, Cluster 1), and eIF3 subunit G (Q1HQN4, FC 48 hpi vs. Control −1.22, FC 48 vs. 12 hpi −1.61, Cluster 1). eIF3 subunits interact with mRNA, eIF4, and the 40S ribosomal subunit. These results suggest that CHIKV induces a protein synthesis shutoff of host proteins in Aag-2 cells, in the same manner as observed before in human cells.

### A Higher Energy Metabolism Demand Is Required for Chikungunya Virus-Infected Aag-2 Cells at 48 hpi

Proteomics revealed that proteins related to energy metabolism processes such as the TCA cycle, glycolysis/gluconeogenesis, and carbon metabolism were more abundant at 48 hpi rather than uninfected and 12 hpi in KEGG enrichment (**Figure 6**). Supporting this observation, STRING PPI analysis showed four *Ae. aegypti* ATP synthesis-related proteins with an elevated abundance at 48 hpi (**Figure 7**). The Rieske subunit of the ubiquinol-cytochrome b-c1 reductase (CIII mitochondrial) (Q17EQ1, FC 48 hpi vs. Control +0.43, Cluster 2) acts by transferring electrons from ubiquinol to cytochrome *c* (cyt *c*) as part of electron transport chain step of oxidative phosphorylation. This protein complex is essential for the respiratory chain functioning (Zara and Conte, 2011), and it allows a proton gradient across the mitochondrial membrane for ATP production by ATP synthase (Shi et al., 2018). The mitochondrial ATP synthase (Q1HR38, FC 48 vs. 12 hpi +0.95, Cluster 3) and ATP synthase gamma subunit (Q16XK3, FC 48 vs. 12 hpi +0.48, Cluster 3) presented an elevated FC at 48 hpi. Considering the comparison of 48 hpi vs. Control, the ATP synthase alpha subunit (Q1HRQ7, FC 48 hpi vs. Control +0.18, Cluster 2) was also more abundant.

Interestingly, two putative mitochondrial RPs were more abundant at 48 hpi. Although mitochondria have derived from endosymbiotic bacteria, mitochondrial ribosomes (mitoribosomes) have strongly diverged in structure and function (Greber and Ban, 2016). Mitoribosomes became specialized in synthesizing membrane proteins, such as important complexes of the mitochondrial respiratory chain (Greber and Ban, 2016). In this study, the putative mitochondrial RP L48 (Q16W93, FC 48 hpi vs. Control +0.86, 48 vs. 12 hpi +0.89) and the putative mitochondrial RP S18A (Q172K6, FC 48 hpi vs. control +0.26) were upregulated at 48 hpi. As long as mitoribosomes are specialized in the mitochondrial protein synthesis, which may include proteins associated with metabolic pathways, this phenomenon could support this higher demand of energy production by host cells for the establishment of CHIKV infection. The mitochondrial metabolic pathway to generate energy on CHIKV-infected Aag-2 cells is overrepresented over 48 h of infection, in agreement with previous CHIKV-infected mosquito cell studies (Lee and Chu, 2015; Vasconcellos et al., 2020; Cui et al., 2020).

Additionally, TEM images exhibited at late-stage infection some mitochondria with an unexpected elongated morphology (**Figure 3F**), as observed in mammalian cells infected with DENV and Zika virus (ZIKV). The same phenomenon was not observed for other *Flaviviridae* members such as West Nile virus (WNV) and hepatitis C virus (HCV) (Chatel-Chaix et al., 2016). In healthy cells, mitochondria form a dynamic net with repeated fission and fusion events that aim to exclude damaged cells by mitophagy, ensuring homeostasis (Tiku et al., 2020). The virus-derived mitochondrial elongation causes an imbalance in this fission and fusion dynamics, allowing damaged or non-efficient mitochondria to stay active in the net (Kim et al., 2017). It has also been observed that infections caused by severe acute respiratory syndrome coronavirus (SARS-CoV) and HIV are also linked to a shift in mitochondrial dynamics toward fusion (elongation), while other viruses cause a shift toward fission (Kim et al., 2017; Tiku et al., 2020). This imbalance could potentially impair energy metabolism and, consequently, lead to glycolysis activation in order to reset energy levels.

In *Influenza*-infected mammalian cells, a mitochondrial membrane disruption with a consequent decrease in ATP levels has been observed, requiring glycolysis activation (Ritter et al., 2010). Moreover, the enhancement of the glycolytic pathway using drugs allowed for higher *Influenza* replication levels (Ren et al., 2021). Other viruses such as DENV, murine norovirus (MNV), human cytomegalovirus (HCMV), and herpes simplex virus 1 (HSV1) also induce increased levels of glycolysis in their host cells (Passalacqua et al., 2019; Thaker et al., 2019). Herein, we suggest that this higher energy demand at a later infection time point could possibly occur due to CHIKV particle production and release, but also due to the consequences of the stress-associated attempt of Aag-2 cells to recover homeostasis. Since energy-related metabolic pathways are host factors that can determine the outcome of a viral infection, they are frequently manipulated by viruses, in different ways, to favor their replication.

### Prohibitins Are Likely Important for Chikungunya Virus Infection in Aag-2 Cells

Prohibitins (PHBs) form a highly evolutionary conserved family of proteins that can act in different cell compartments, such as the plasmatic membrane, nucleus, and inner mitochondrial membrane, having specific site-associated functions (Mishra et al., 2006; Merkwirth et al., 2007). Moreover, these proteins are ubiquitously expressed in animals, plants, and fungi (Chowdhury et al., 2014). In this study, two PHBs were upregulated at 48 hpi: A0A6I8TPE2 (FC 48 hpi vs. Control +0.42), which is generically classified as membranous in the UniProt database, and Q1HR13 (FC 48 hpi vs. Control +0.28). The second PHB is classified as a mitochondrial inner membrane protein in the UniProt database. In addition, it is also listed as an overrepresented mitochondrion gene set (orange) of the STRING PPI analysis when comparing 48 hpi vs. Control (**Figure 7A**). Mitochondrial PHBs have an important role in stabilizing the dynamin-related OPA1, a protein that promotes mitochondrial fusion (Merkwirth et al., 2007; Ban et al., 2017). These PHBs also contribute to the electron chain transport activity, to maintain the integrity of the mitochondrial inner membrane (Foo et al., 2021). Furthermore, previous studies on arboviruses have identified surface PHB as a receptor for DENV entrance in *Ae. aegypti* and *Ae. albopictus* cells. The same mechanism is observed for CHIKV concerning different mammalian cell types (Hidari and Suzuki, 2011; Wintachai et al., 2012). A previous study from our group also showed that PHB abundance from MAYV-infected Aag-2 cells was increased over 48 hpi (Vasconcellos et al., 2020). Overall, PHBs could have two relevant roles for CHIKV infection in *Ae. aegypti* cells: to act as a surface receptor, allowing viral entrance, and to support the inner mitochondrial membrane and mitochondrial fusion. These mechanisms could explain the elongated mitochondrial morphology observed in the 48-hpi TEM image (**Figure 3F**).

### Mitochondrial Elongation May Be Related to Later Apoptosis During Chikungunya Virus Infection

In addition to bioenergetic metabolism, mitochondria also play a central role in innate immune signaling and cell survival (Kim et al., 2017). The fission and fusion imbalance possibly caused by the virus in the mitochondrial net can also impair host homeostasis and progression of the cell cycle, leading to apoptosis activation (Tiku et al., 2020). In a viral replication cycle, early activation of programmed cell death may not be advantageous if higher amounts of viral particles are ready to be released during later infection time points. Thus, postponing host cell death is a signal of a well-succeeded infection (Datan et al., 2016). It has been reported that during CHIKV infection, reactive oxygen species (ROS)-activated autophagy mediates a signaling cascade that delays apoptotic cell death in mammalian cells (Joubert et al., 2012), a scenario that benefits viral propagation. While DENV and ZIKV also manipulate host autophagy to improve their replication levels (Datan et al., 2016), apoptotic blebs containing CHIKV particles have been observed (Joubert et al., 2012). Furthermore, proteins of viruses such as HCV and HIV have been shown to interact with host mitochondrial membranes to increase ROS production and benefit viral replication (Foo et al., 2021).

Two significant cell sites for ROS production are the mitochondrial Complexes I and III of the electron transport chain (Chen et al., 2003; Foo et al., 2021). In the present study, the Aag-2 Rieske subunit of the ubiquinol-cytochrome b-c1 reductase (CIII) mitochondrial complex (Q17EQ1, FC 48 hpi vs. Control +0.43, Cluster 2) was more abundant at 48 hpi rather than at 12 hpi and Control. This complex acts by transferring electrons to cyt *c*, another compound well known to trigger apoptosis (Jiang and Wang, 2004). Moreover, cyt *c* also has dual functions in energy metabolism and cell death (Cai et al., 1998). When released from mitochondria, cyt *c* interacts with the apoptotic protease activating factor 1 (Apaf1) to form a complex that activates caspase 9, which is cleaved into caspases 3 and 7 (Bratton and Salvesen, 2010). Then, this cyt *c*-dependent release of the caspases 3 and 7 triggers protein degradation and apoptosis in host cells (Jiang and Wang, 2004). In this study, we propose that CHIKV might create a mitochondrial net imbalance in *Ae. aegypti* cells to delay apoptosis in a ROS-dependent fashion and, consequently, earn some proliferation time.

### Different Aag-2 RNA Helicases Were Modulated

We have observed 8 host RNA helicases that presented a differential abundance in the proteomic dataset. Three of them were upregulated over time points: Q0IEJ1 (FC 12 hpi vs. Control +1.19, Cluster 2), A0A1S4EZS3 (FC 12 hpi vs. Control +0.55 and FC 48 hpi vs. Control +0.66, Cluster 2), and Q178X5 (FC 12 hpi vs. Control +0.49 and FC 48 hpi vs. Control +0.69). The rest of them were downregulated over time points: A0A1S4F0V4 (FC 12 hpi vs. Control −0.43 and FC 48 hpi vs. Control −0.71), Q16XX4 (FC 48 hpi vs. Control −0.62), Q16RY3 (FC 48 hpi vs. Control −0.81 and FC 48 vs. 12 hpi −0.48, Cluster 1), A0A6I8TAB4 (FC 48 vs. 12 hpi −0.28, Cluster 1), and Q17CT5 (FC 48 vs. 12 hpi −0.91) (**Supplementary Table 2**). In agreement with UniProt database predictions, six RNA helicases (Q0IEJ1, A0A1S4EZS3, Q178X5, A0A1S4F0V4, Q16XX4, and Q16RY3) presented three conserved motifs: Q-motif, helicase ATP-binding, and helicase C-domain. The remaining two RNA helicases (A0A6I8TAB4 and Q17CT5) have no Q-motif. From these predictions, it was also observed that all RNA helicases in our study are classified as members of the DEAD/DEAH box helicases family (Schmid and Linder, 1992; Tanner and Linder, 2001; Caruthers and McKay, 2002; Tanner et al., 2003). Recent reports have shown that DEAD/DEAH box RNA helicases are also important for the recognition of foreign nucleic acids and modulation of viral infection, possibly acting as sensors for innate immune mechanisms that will influence viral replication (Fullam and Schröder, 2013; Taschuk and Cherry, 2020; Ali, 2021).

Frequently, DEAD/DEAH box RNA helicases are also referred to as DDX (Fullam and Schröder, 2013; Ali, 2021). For hepatitis B virus (HBV) in human cells, DDX3 binds to HBV reverse transcriptase, activating a signaling cascade that impairs IFN-regulatory factors (IRF) and IFN-β promoter activity, facilitating viral replication (Wang and Ryu, 2010). However, at later infection stages, DDX3 is required to contain HBV reverse transcription, therefore hampering its replication (Wang et al., 2009). These proviral and antiviral patterns related to DDX are similarly found during infection by other arboviruses. *Ae. aegypti* DDX6 has antiviral properties against ZIKV and WNV. In both cases, DDX6 binds to the 3′ UTR subgenomic flavivirus RNA (sfRNA), inhibiting viral replication in Aag-2 cells (Göertz et al., 2019). During DENV infection, DDX3X supposedly interacts with viral capsid to inhibit DENV replication (Kumar et al., 2018). For CHIKV infection, the *Ae. aegypti* DEAD-box helicase RM62F was found to interact with the viral protein nsP3 in Aag-2 cells, leading to suppression of RNAi pathway for gene silencing (Kumar et al., 2021). It is noteworthy to mention that the interfering RNA mechanism for post-transcriptional gene silencing is an antiviral conserved hallmark for hosts such as nematodes, insects, fungi, and plants (Chang et al., 2012; Gammon et al., 2017; Vogel et al., 2019; Schuster et al., 2019).

Overall, different classes of host DEAD/DEAH box RNA helicases can contribute to immune-modulatory mechanisms or even be hijacked by viruses to support their replication. Therefore, these proteins may act in a proviral fashion and as antiviral effectors (Ali, 2021). It is tempting to suppose that the 8 DEAD/DEAH box RNA helicases that displayed a differential abundance could support both cellular mechanisms in CHIKV-infected Aag-2 cells. Possibly, a preparation phase for CHIKV viral particle production before programmed cell death would require the three DEAD/DEAH box RNA helicases found as upregulated in our dataset (Q0IEJ1, A0A1S4EZS3, and Q178X5). However, once CHIKV infection is established, the five remaining downregulated RNA helicases (A0A1S4F0V4, Q16XX4, Q16RY3, A0A6I8TAB4, and Q17CT5) could reflect a suggestive inhibition strategy of antiviral mechanisms, such as RNAi, to support viral replication. In fact, it has been already shown that Ago2, a component of the RNAi machinery, was downregulated at 48 hpi in CHIKV-infected Aag-2 cells (Kumar et al., 2021). Since we have observed a significant count of viral particles in the Aag-2 cells at 48 hpi (**Figure 3G**) and an increasing abundance percentage of CHIKV peptides throughout the infection (**Figure 4**), we suggest that DEAD/DEAH box RNA helicases could be involved in both pro- and antiviral host strategies to promote the propagation.

### Conclusions

The present report brings an analysis of the molecular aspects of *Ae. aegypti* cells infected with CHIKV, by which 196 Aag-2 proteins were modulated upon CHIKV infection. Mainly, our study has shown that these host proteins are associated with protein synthesis, signaling pathways, energy metabolism, and apoptosis. The notorious viral count produced by mosquito cells over 48 h observed by our TEM analysis probably has influenced the host energy metabolism, in order to produce more ATP, as revealed by the label-free quantitative proteomic analysis. It would be interesting to study if glycolysis in Aag-2 cells could be activated upon viral infection to increase cell ATP levels and sustain virus replication, by analyzing the effect of glycolysis inhibitors, such as 2-deoxy-d-glucose (2DG). Moreover, the mitochondrial elongated morphology observed at 48 hpi may reflect a mitochondrial net imbalance that could affect energy metabolism and regulate apoptosis timing. It is widely accepted that different virus species modulate mitochondrial bioenergetics to enhance viral replication. As part of a central role in the viral replication and oxidative stress, the (dys)function of host mitochondria seems to be an essential element for many viruses (El-Bacha and Da Poian, 2013). For instance, an enhanced level of ROS inhibits DENV infection (Khan et al., 2021). In the ZIKV, this ROS imbalance is associated with mitochondrial and DNA damage in human astrocytes (Ledur et al., 2020). In both cases, the use of fluorescent probes against free radicals, such as 2′,7′-dichlorodihydrofluorescein diacetate (DCFDA) and dihydroethidium (DHE), were respectively used (Ledur et al., 2020; Khan et al., 2021). Perhaps novel studies regarding free radicals production upon CHIKV infection could cast a glance on whether the same phenomena could take place in Aag-2 insect cells. Based on what is observed in other vector-borne viruses, these classes of proteins may be acting in a pro- and antiviral fashion to support the balance between viral propagation and the survival inside host cells, leading to the persistent infection. Additionally, metabolomics studies would provide complementary information regarding subtle metabolic shifts (Tounta et al., 2021). Overall, our data may contribute to a better comprehension of the adaptive molecular mechanisms of *Ae. aegypti* concerning CHIKV infection.

## Data Availability Statement

The datasets presented in this study can be found in online repositories. The name of the repository and accession number can be found below: ProteomeXchange via the PRIDE (Perez-Riverol et al., 2018) database; PXD033231.

## Author Contributions

AV, SM, AO, SC, and RR designed the study. AV and AO performed the infection experiments. SC and RR supervised the experiments. SM, EM, and MT performed the LC-MS/MS analysis. RM performed the bioinformatics analysis. AV, RM, LO, and SC interpreted the data, designed the figures, and wrote the manuscript. SB performed electron microscopy. All authors revised and approved the submitted version.

## Funding

This work was supported by grants and fellowships awarded by the Fundação de Amparo à Pesquisa do Distrito Federal (FAP-DF, 0193.000417/2016 and 00193-00000779/2021-40), Coordenação de Aperfeiçoamento de Pessoal de Nível Superior (CAPES, 23038.013502/2020-21), Conselho Nacional de Desenvolvimento Científico e Tecnoloígico (CNPq, INCT-MCTI/CNPq/FAPs 16/2014), and Fiocruz (INOVA Program). The funders had no role in study design, data collection and interpretation, or the decision to submit the work for publication.

## Conflict of Interest

The authors declare that the research was conducted in the absence of any commercial or financial relationships that could be construed as a potential conflict of interest.

## Publisher’s Note

All claims expressed in this article are solely those of the authors and do not necessarily represent those of their affiliated organizations, or those of the publisher, the editors and the reviewers. Any product that may be evaluated in this article, or claim that may be made by its manufacturer, is not guaranteed or endorsed by the publisher.

## Supplementary Material

Supplementary Figure 1 Optical microscopy of infection tests using Vero cells. Vero cells were mock-infected **(A)** and infected with Dengue virus 2 (DENV2) **(B)**, Mayaro virus (MAYV) **(C)** and Chikungunya virus (CHIKV) **(D)**, separately, using MOI 1. Visually, the CHIKV isolate was more virulent, leading to a higher degree of cytopathic effect (formation of syncytia and cell death) than DENV2 and MAYV isolates. Vero cells are fixed in culture and produce an extracellular matrix that is degraded after cell death. Then, cells become rounded.

Supplementary Figure 2 Quality control of protein group identification and quantification. **(A)** Boxplot of log_2_ protein group abundances. **(B)** Histogram of log_2_ protein groups abundances. **(C)** Number of valid values according to the number of replicates, cut-off of three replicates was used in this analysis, represented by the dashed line. **(D)** filter for ANOVA and g-test analysis, only ANOVA filter was used, set by two replicates per group, represented by the dashed line. **(E)** robust Mahalanobis distance analysis considering Correlation, median absolute deviation, skewness and proportion of missing values, suggesting the absence of extreme outliers (p-value = 1e-04). **(F)** probabilistic principal component analysis of protein group abundances according to each replicate.

Supplementary Figure 3 Bubble plot of enriched biological process GO terms in VSclust clusters. GO terms enrichment values are represented by colored circumferences. A clear predominance of translation GO terms can be seen in the enriched terms of Cluster 1, mainly in those with eneRatio values.
