# Supplementary figures and images for: Aedes aegypti Aag-2 Cell Proteome Modulation in Response to Chikungunya Virus Infection
Source: Front Cell Infect Microbiol. 2022 Jun 15;12:920425. doi: 10.3389/fcimb.2022.920425 (PMC9240781; doi:10.3389/fcimb.2022.920425)

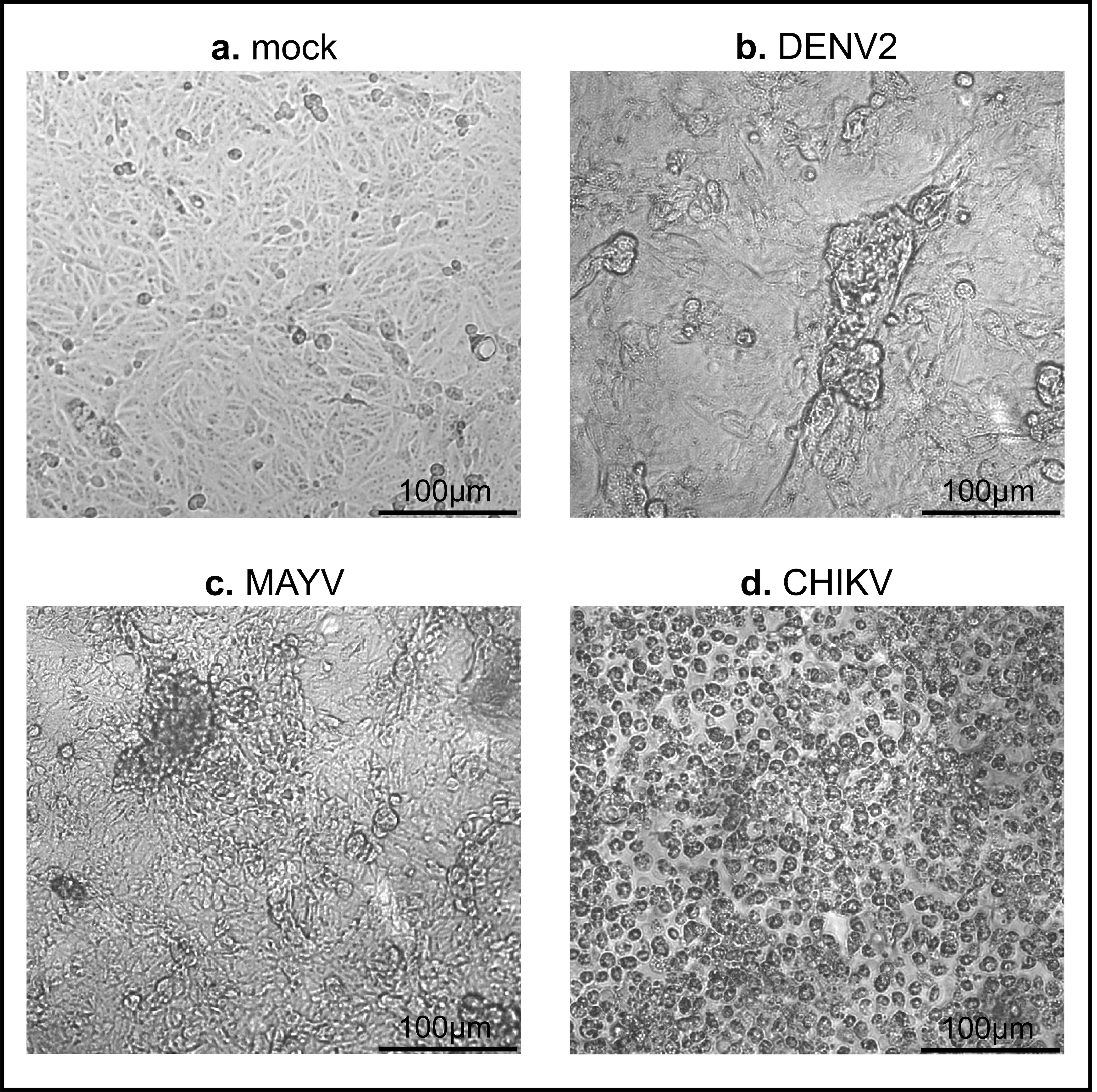

Supplement: Supplementary Figure 1 — Optical microscopy of infection tests using Vero cells. Vero cells were mock-infected (A) and infected with Dengue virus 2 (DENV2) (B), Mayaro virus (MAYV) (C) and Chikungunya virus (CHIKV) (D), separately, using MOI 1. Visually, the CHIKV isolate was more virulent, leading to a higher degree of cytopathic effect (formation of syncytia and cell death) than DENV2 and MAYV isolates. Vero cells are fixed in culture and produce an extracellular matrix that is degraded after cell death. Then, cells become rounded. [file Image_1.tif]

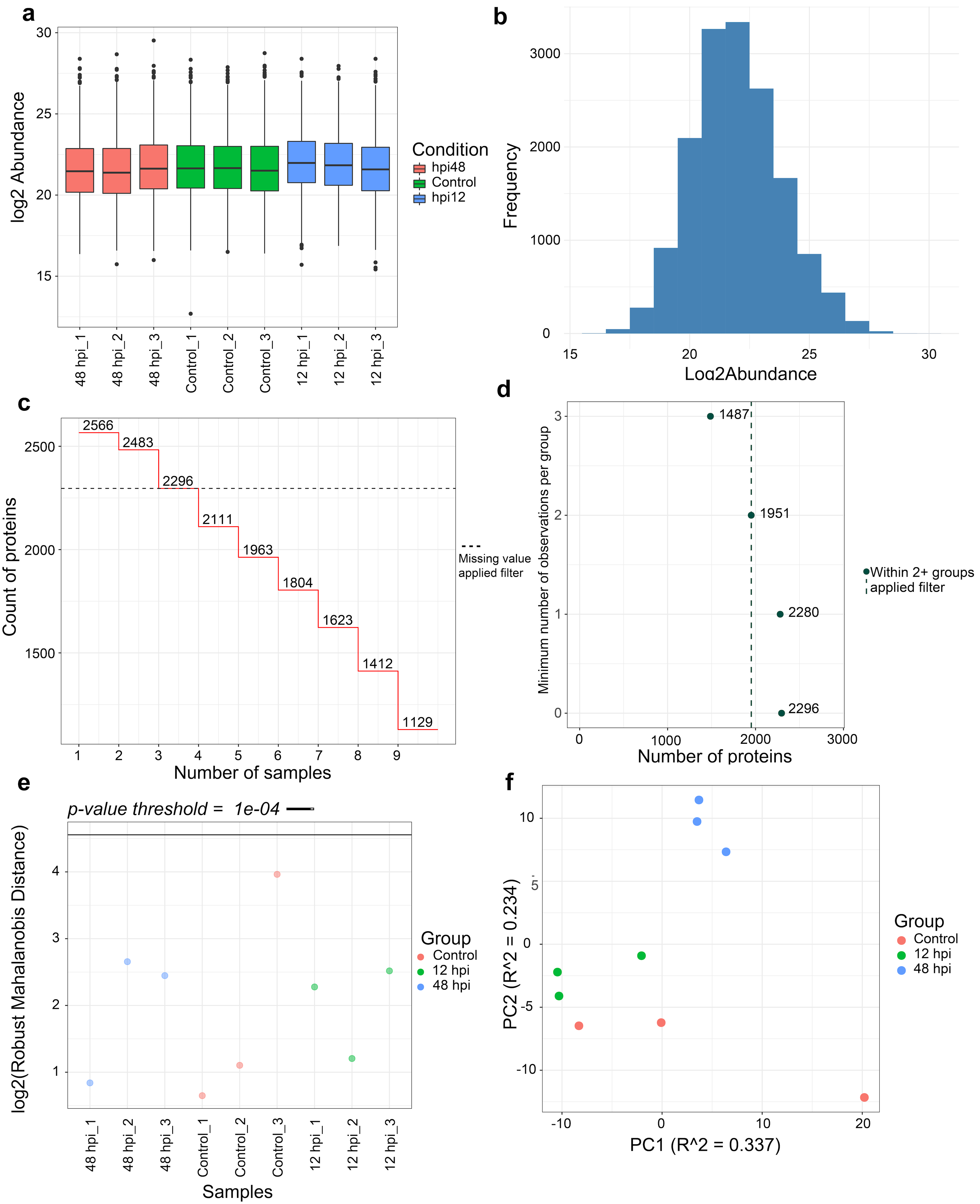

Supplement: Supplementary Figure 2 — Quality control of protein group identification and quantification. (A) Boxplot of log2 protein group abundances. (B) Histogram of log2 protein groups abundances. (C) Number of valid values according to the number of replicates, cut-off of three replicates was used in this analysis, represented by the dashed line. (D) filter for ANOVA and g-test analysis, only ANOVA filter was used, set by two replicates per group, represented by the dashed line. (E) robust Mahalanobis distance analysis considering Correlation, median absolute deviation, skewness and proportion of missing values, suggesting the absence of extreme outliers (p-value = 1e-04). (F) probabilistic principal component analysis of protein group abundances according to each replicate. [file Image_2.tif]

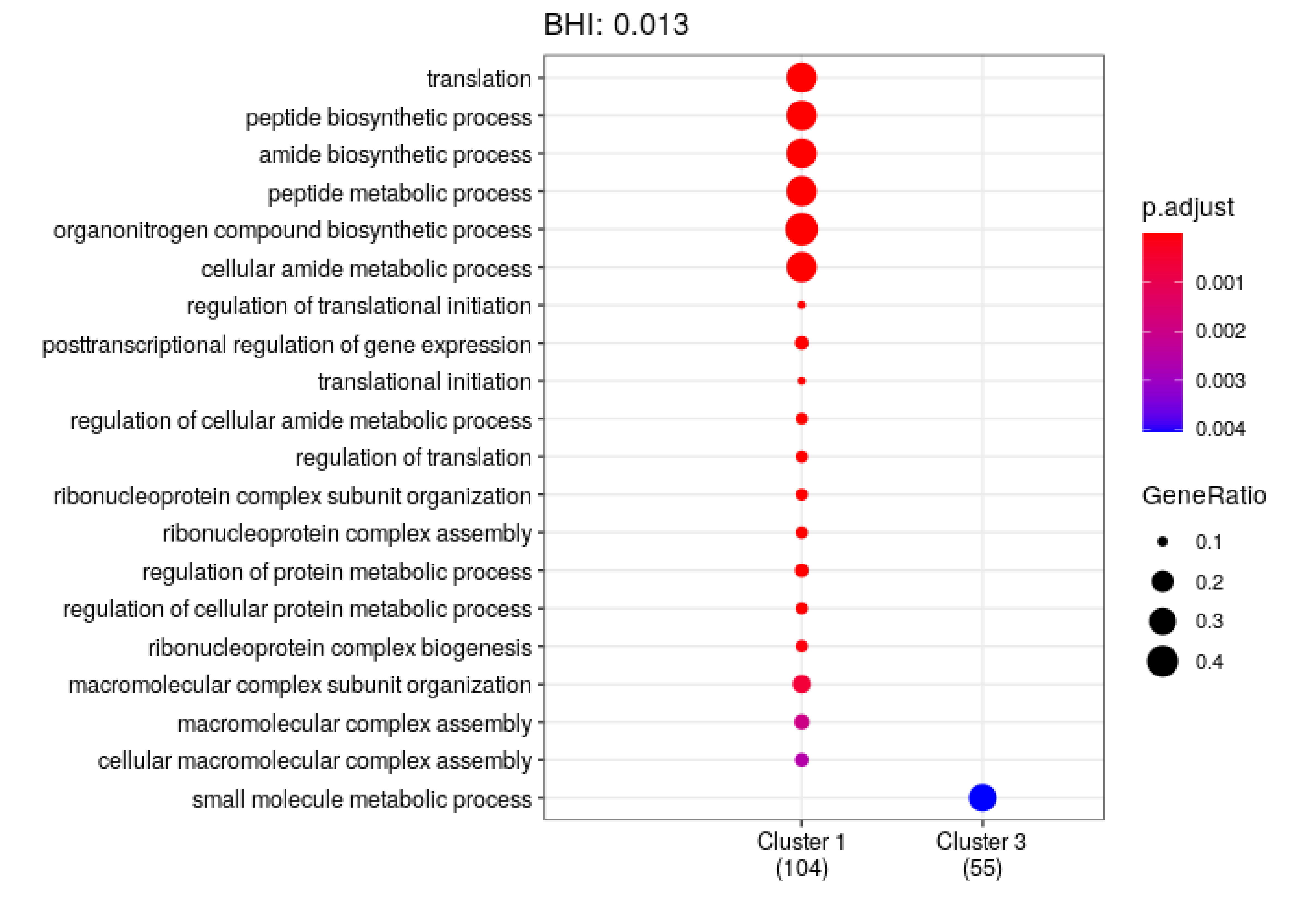

Supplement: Supplementary Figure 3 — Bubble plot of enriched biological process GO terms in VSclust clusters. GO terms enrichment values are represented by colored circumferences. A clear predominance of translation GO terms can be seen in the enriched terms of Cluster 1, mainly in those with eneRatio values. [file Image_3.tif]
